# Differentiation enhances aminolevulinic acid-dependent photodynamic treatment of LNCaP prostate cancer cells

**DOI:** 10.1038/sj.bjc.6600575

**Published:** 2002-11-12

**Authors:** B Ortel, D Sharlin, D O'Donnell, A K Sinha, E V Maytin, T Hasan

**Affiliations:** Wellman Laboratories of Photomedicine WEL-224, Department of Dermatology, Massachusetts General Hospital, Harvard Medical School, 55 Fruit Street, Boston, Massachusetts, MA 02114, USA; Department of Biomedical Engineering ND-20, Lerner Research Institute, The Cleveland Clinic Foundation, 9500 Euclid Avenue, Cleveland, Ohio, OH 44195, USA

**Keywords:** photodynamic therapy, retinoid, aminolevulinic acid, vitamin D, androgen

## Abstract

Photodynamic therapy using 5-aminolevulinic acid (ALA)-induced protoporphyrin IX (PpIX) may be applied to the treatment of neoplasms in a variety of organs. In order to enhance existing regimens of photodynamic therapy, we investigated the effects of adding differentiation therapy to photodynamic therapy in human prostate cancer cells *in vitro*. The objective of differentiation therapy *per se* is to reverse the lack of differentiation in cancer cells using pharmacological agents. The motivation for this study was to exploit the differentiation-dependent expression of some heme enzymes to enhance tumour cell toxicity of ALA-photodynamic therapy. A short course of differentiation therapy was applied to increase PpIX formation during subsequent ALA exposure. Using the synthetic androgen R1881, isomers of retinoic acid, and analogues of vitamin D for 3 to 4 days, exogenous ALA-dependent PpIX formation in LNCaP cells was increased, along with markers for growth arrest and for differentiation. As a consequence of higher PpIX levels, cytotoxic effects of visible light exposure were also enhanced. Short-term differentiation therapy increased not only the overall PpIX production but also reduced that fraction of cells that contained low PpIX levels as demonstrated by flow cytometry and fluorescence microscopy. This study suggests that it will be feasible to develop protocols combining short-term differentiation therapy with photodynamic therapy for enhanced photosensitisation.

*British Journal of Cancer* (2002) **87**, 1321–1327. doi:10.1038/sj.bjc.6600575 www.bjcancer.com

© 2002 Cancer Research UK

## 

Photodynamic therapy (PDT), the administration of an exogenous photosensitizer plus light, is becoming increasingly recognised as a viable alternative to other cancer therapies (Hasan *et al*, 2000). Recently, a newer approach to PDT has attracted much interest, namely, the application of a pro-drug (5-aminolevulinic acid, ALA) that is converted *in situ* into a photosensitizing compound (protoporphyrin IX, PpIX) (Peng *et al*, 1997). While ALA-mediated PDT has shown some promise in pilot studies of certain malignancies to date, including cancers of the skin (Svanberg *et al*, 1994; Fijan *et al*, 1995; Peng *et al*, 1997), the number of treatment failures remains unacceptable. While a variety of mechanisms may lead to these treatment failures, all failures by definition involve a subpopulation of cancer cells that manage to escape cell death. One promising and relatively unexplored approach to improving the efficacy of cell-killing is to alter biological responses of the target cancer cells, in a manner that enhances susceptibility to ALA-mediated PDT. One example of such an approach is the use of short-term differentiation therapy.

Differentiation therapy (DT) makes use of the fact that cancer cells often do not progress through the normal processes of growth arrest, differentiation, and scheduled apoptosis, and thus circumvent cell death (Andreeff *et al*, 2000). To counteract these abnormalities, pharmacological agents are applied to redirect aberrant pathways, so that cancer cells mature and eventually undergo normal apoptosis. A familiar clinical DT paradigm is the use of retinoic acid, administered over several months in patients with promyelocytic leukemia (APML) (Fenaux *et al*, 2001). DT causes abnormal promyelocytes to differentiate, becoming segmented granulocytes with a limited lifetime (Glasser *et al*, 1994). Our approach to DT differs from the traditional definition of DT in two respects: (1) it exploits the fact that a photosensitising porphyrin intermediate, PpIX, can accumulate to high levels preferentially in differentiated cells (see below); (2) the differentiation-modulating agent is administered over a relatively short period of time (days) to prepare the cells for a second therapeutic modality, namely exposure to ALA and light.

The approach described here arose out of an exploration of the capacity of cells to synthesise PpIX (the photosensitiser) from ALA (the pro-drug), and is based upon the finding that cellular differentiation increases the ability of a variety of cells to synthesise PpIX from exogenous ALA (Ortel *et al*, 1998). For example, induction of differentiation in skin keratinocytes (by increasing calcium concentrations in the culture medium) leads to an accumulation in PpIX, involving upregulation of the heme-synthetic enzyme *coproporphyrinogen oxidase*, and results in significantly increased photosensitisation (Ortel *et al*, 1998). Here, we evaluate a similar approach, but now using DT and ALA-PDT for human prostate cancer cells. Previous work by our group used the LNCaP cell line in PDT studies (Momma *et al*, 1998; Hamblin *et al*, 1999) and showed that ALA-induced PpIX formation can be manipulated by androgenic hormones (Momma *et al*, 1997). Our current work extends that concept by showing that other agents can also alter PpIX levels when used in a short-term course for DT. Furthermore, we demonstrate that DT enhances lethal photosensitisation. The data suggest that combination regimens consisting of DT using vitamin D or its analogues, followed by cell-killing with ALA-PDT, may represent a useful therapeutic approach to the management of prostate cancer.

## MATERIALS AND METHODS

### Cell culture conditions

Human LNCaP cells were obtained from American Type Culture Collection (Rockville, MD, USA) and grown in RPMI 1640 (Mediatech, Inc., Herndon, VA, USA) supplemented with antibiotics, 10 mm HEPES, and 10% foetal calf serum (FCS, Gibco, Invitrogen Corporation, Carlsbad, CA, USA) at 37°C in a humidified atmosphere of 5% CO_2_. All experiments were done using duplicate samples and at least three different times.

### Chemicals

ALA was obtained from Sigma (St. Louis, MO, USA), R1881 (methyltrienolone) was from PerkinElmer Life Sciences (Boston, MA, USA). Vitamin D (in the form of calcitriol), and its analogues RO-26-2198 and RO-25-9022 were synthesised and previously described by Dr Milan Uskokovic (Hofmann LaRoche, Nutley, NJ, USA), who provided them as generous gifts. All-trans-, 9-cis-, and 13-cis-retinoic acid were from Sigma.

### Differentiation therapy

For DT, cells were plated at a density of 7.5 to 15×10^4^ cells per 35 mm petri dish. After 24 h the medium was replaced with medium supplemented with delipidised 10% foetal calf serum (Cocalico Biologicals, Inc., Reamstown, PA, USA). The differentiating agents were diluted into pre-warmed medium and immediately added to the cells. Controls included medium with delipidised FCS and delipidised FCS plus vehicle. All agents were used as a single pulse dose of 72 h duration.

### DNA synthesis assay

Cells (3–4×10^5^ per 35 mm dish) were incubated with 1 μCi ml^−1^ [*methyl*-^3^H]thymidine (20.0 Ci mmol^−1^ from Dupont/NEN, Wilmington, DE, USA) in 0.5 ml media. After 4 h DNA was isolated by acid precipitation and tritium quantified as described previously (Alge *et al*, 2001).

### Western blotting

Whole cell lysates were obtained by dissolving cells in gel loading buffer, separated on 10 or 12% polyacrylamide gels and transferred onto a Immobilon-P membrane (Millipore, Bedford, MA, USA). E-cadherin and p27/kip-1 were detected using monoclonal mouse antibodies directed against human E-cadherin or human p27 (Santa Cruz Biotechnology, Santa Cruz, CA, USA). A horseradish peroxidase-coupled goat antibody against mouse immunoglobulin (Bio-Rad Laboratories, Hercules, CA, USA) was used for chemiluminescence detection.

### PpIX production and quantification

Cells in 35 mm dishes were incubated in duplicate in 1 ml medium containing 0.1 to 1.0 mM ALA. All manipulations of ALA-treated cells were performed under reduced light conditions. After 4 h, samples were used for PpIX quantification as previously described (Ortel *et al*, 1998). In brief, cells were solubilised in 1% SDS in 0.1 N NaOH and submitted to quantitative spectrofluorometry (excitation 400 nm, emission 580–720 nm, peak 630 nm).

### Fluorescence analysis in living cells

Fluorescence of living cells was analysed by fluorescence microscopy and by flow cytometry. Cells were plated and treated identically to the conditions used to quantify PpIX. For fluorescence microscopy, cells were grown on glass cover slips. A Leica confocal laser scanning microscope (Leica Mikroskopie und System GmbH, Wetzlar, Germany) was used as described in detail earlier (Pogue *et al*, 2001). In brief, the fluorescence signal was separated into a green (525–550 nm) and a red (>590 nm) portion. Fluorescence images were displayed in green and red false colour and electronically overlaid. Fluorescence of individual cells was quantified using flow cytometry (Facscalibur, Beckton Dickinson). ALA-treated cells were incubated for 30 s in trypsin/EDTA (0.25/0.1%) and resuspended after addition of complete medium. Cell suspensions were put on ice and analysed without delay. Using excitation at 488 nm, red fluorescence emission of 10 000 cells was recorded through a 670 nm longpass filter.

### Photosensitisation

Cells (3–5×10^5^ per 35 mm dish) were incubated with 0.3 mM ALA for 4 h and then exposed from below to the expanded homogenous beam of 514 nm Argon laser radiation (Coherent, Inc., Santa Clara, CA, USA) at a dose rate of 0.06 to 0.075 Wcm^−2^ as measured by a Coherent Lasermate power meter.

### MTT survival assay

Quantification of cellular dehydrogenase activity provides a sensitive way of assessing survival after PDT and has been shown to correlate well with other established measures of cytotoxicity such as colony formation (Iinuma *et al*, 1994). The MTT assay was performed at 24 h after light exposure as described in detail earlier (Ortel *et al*, 1998).

### Clonogenic assay

After ALA-PDT as described above, LNCaP cells were detached using trypsin/EDTA and resuspended in complete medium. Diluted suspensions (range 1 : 5 to 1 : 1375) were plated on 100 mm dishes and incubated for 13 days. Cells were fixed with 0.2% buffered formalin in methanol and stained with 0.1% aqueous crystal violet. Colonies of more than 50 cells were counted under a dissecting microscope.

### Statistical analysis

The *t*-test for comparison of means or the paired *t*-test was used for statistical analysis. *P*-values less than 0.05 were considered statistically significant.

## RESULTS

### Androgen treatment of LNCaP cells arrests growth and induces differentiation

**Figure 1:**
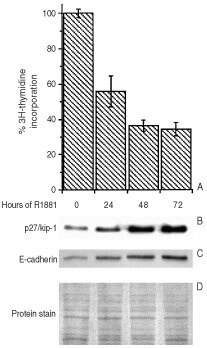
Growth arrest and differentiation in LNCaP cells. Treatment with 10^−7^ M R1881 suppressed the growth of LNCaP cells as documented by (**A**) reduced incorporation of tritiated thymidine. Western blotting (**B**,**C**) demonstrated (**B**) upregulation of the cell cycle inhibitor p27/Kip-1 and the differentiation marker E-cadherin (**C**) after R1881 treatment. (**D**) Protein staining with Coomassie blue served as loading control.

Exposure of LNCaP cells to 10^−7^ M R1881 resulted in reduced DNA synthesis by 48–72 h (^3^H-thymidine incorporation, Figure 1A

). Simultaneously, p27/Kip 1, an inhibitor of cyclin-dependent kinases whose upregulation correlates with growth-arrest, was increased at the protein level up to 3.4 times as shown by Western blotting (Figure 1B). Furthermore, R1881 treatment resulted in a more than two-fold rise in E-cadherin protein expression (Figure 1C), a generally-accepted indicator of differentiation in LNCaP cells (Campbell *et al*, 1999).

### Androgen treatment of LNCaP cells increases ALA-induced PpIX production

**Figure 2:**
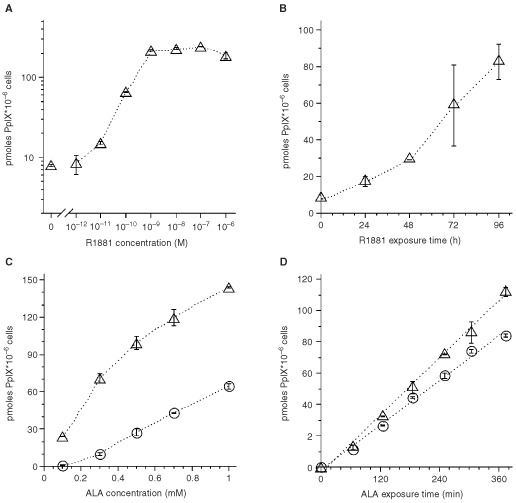
Increased ALA-induced PpIX production in cells treated with R1881. Pretreatment variables were (**A**) R1881 concentration (exposure for 72 h) and (**B**) R1881 incubation time (at 10^−7^ M). (**C**) At all ALA concentrations, R1881(10^−7^ M)-pretreated cells (▵) accumulated higher PpIX amounts than control cells (O). (**D**) When incubating differentiated cells with 0.2 mM ALA (▵) and undifferentiated cells with 0.6 mM ALA (O), respectively, there was a linear increase in PpIX content in both sets of cells. Experiments show mean±s.d. values of duplicate samples.

When LNCaP cells were pretreated with 10^−7^ M R1881 for 72 h, subsequent exposure to 0.3 mM ALA induced 28.6±27.2-fold higher PpIX accumulation in androgen-pretreated cells than in proliferating control cells (13 experiments, two dishes/experiment). This effect depended on R1881 dose and exposure time (Figure 2A,B

). Androgen-pretreated cells produced at least twice the amount of PpIX as control cells at all concentrations up to 1 mM (Figure 2C). Androgen-differentiated and control cells were also treated with different concentrations of ALA to reach similar PpIX formation. In this setting, both samples showed very similar and linear increase of PpIX content (Figure 2D).

### Androgen pretreatment increases ALA-induced phototoxicity

**Figure 3:**
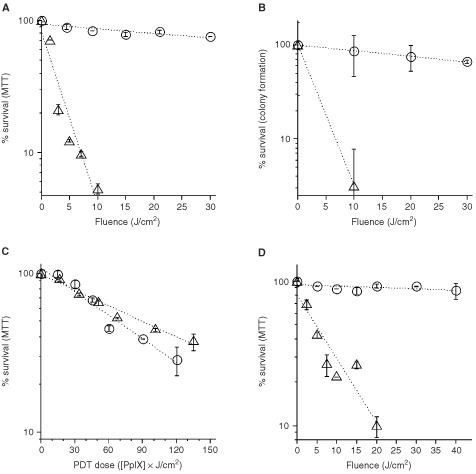
Photosensitisation of DT-exposed cells. LNCaP cells pretreated for 72 h with 10^−7^ M R1881 (▵) and control cells (O) were incubated for 4 h with 0.3 mm ALA and irradiated. PpIX-mediated phototoxicity was significantly enhanced in androgen-pretreated cells (▵) using (**A**) MTT conversion and (**B**) colony formation as endpoints. Differentiated (▵) and control cells (O) containing similar amounts of PpIX (**C**) showed a small decrease in photosensitivity in the differentiated cells. (**D**) Pretreatment with 10^−7^ M Ro-26-2198 for 96 h before photosensitisation resulted in increased cytotoxicity in Ro-26-2198-treated cells (▵) as compared to control cells (O), as assessed by MTT conversion. All data points are mean±s.d. values of duplicate samples.

R1881-treated (10^−7^ M, 72 h) and control LNCaP cells were incubated with 0.3 mM ALA for 4 h and then exposed to 514 nm radiation. R1881-treated cells were killed at much lower fluences than undifferentiated cells (Figure 3A

). The increase in phototoxicity was shown to be statistically significant using MTT conversion (Figure 3A). The light dose requirements were calculated from the slopes of the survival curves. There was a 58±28-fold dose ratio of the irradiation required to reduce survival to 50% in differentiated *vs* undifferentiated cells. The evaluation of colony formation as endpoint of phototoxicity also showed a highly significant increase in phototoxicity in R1881-treated cells (Figure 3B).

### Androgen pretreatment does not inherently increase phototoxic efficiency

In order to compare phototoxicity at equal cellular PpIX concentrations, undifferentiated cells were treated with 0.6 mM ALA while R1881-treated cells received 0.2 mM ALA. Both sets of cells produced similar amounts of PpIX (Figure 2C,D). Using these conditions, we compared the phototoxic efficacy (survival as a function of the combined PpIX concentration and irradiation dose). There was a shift of the survival curve of the R1881-treated cells indicating increased resistance to PDT as compared to undifferentiated cells (Figure 3C). This difference was small but statistically significant (*P*<0.05).

### Androgen treatment increases the fraction of cells with high PpIX concentration

**Figure 4:**
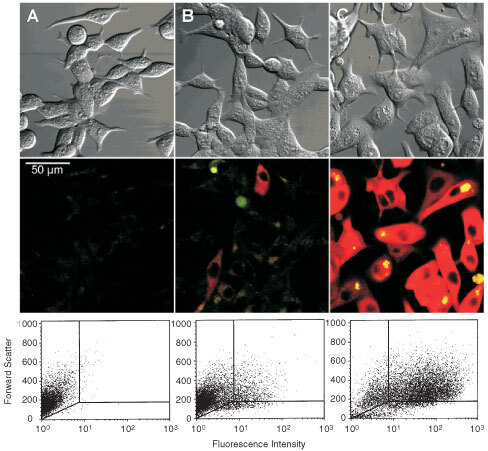
PpIX fluorescence in living cells. LNCaP cells were pretreated with vehicle (**A**,**B**) or with 10^−7^ M R1881 for 72 h (**C**). Microscopy. Transmission and fluorescence images of untreated LNCaP cells (**A**) and cells exposed to 0.3 mm ALA (**B**,**C**) without (**B**) or with (**C**) pretreatment with R1881. Flow cytometry. A large fraction of 0.3 mM ALA-treated control cells (**B**) did not show more intense fluorescence than cells (**A**) that were not exposed to ALA. Pretreatment with R1881 (10^−7^ M for 72 h) (**C**) resulted in strongly increased fluorescence in the vast majority of cells.

R1881-treated cells not only showed higher average concentrations of PpIX than control cells (Figure 2), but also contained a larger fraction of cells that emitted strong fluorescence, indicative of high PpIX concentrations. Figure 4

shows transmission and fluorescence microscopy, and flow cytometry histograms to illustrate this point. Vehicle-treated controls incubated with ALA (Figure 4B) showed a large fraction of cells that displayed the same low red fluorescence intensities as untreated cells without ALA incubation (Figure 4A). In R1881-pretreated, ALA-treated samples (Figure 4C) the vast majority of cells showed strong fluorescence. More than 80% of the androgen-pretreated cells exhibited strong cellular red fluorescence, while the majority of vehicle-treated control cells yielded background fluorescence levels.

### Differentiation therapy with vitamin D and its derivatives increases ALA-induced PpIX and ALA*-*PDT

**Figure 5:**
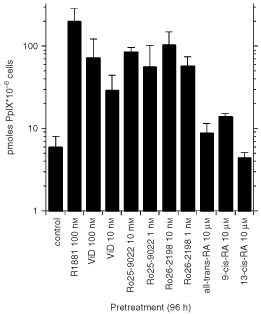
Increased PpIX accumulation after treatment with differentiating agents. LNCaP cells were pretreated with vehicle, retinoids, vitamin D, or vitamin D analogues for 96 h. R1881 (10^−7^ M) served as positive control. Vitamin D and the analogues Ro-25-9022 and Ro-26-2198 strongly enhanced PpIX formation. Even 1000-fold higher doses of the retinoids showed only moderate or no increase in ALA-dependent PpIX formation. Values represent mean+s.d. of 3–8 samples.

Although R1881 is a useful agent *in vitro*, it is unlikely that androgens will be used for prostate cancer in a clinical setting. Therefore we investigated the effect of several differentiation-inducing agents that may be useful *in vivo*. We tested vitamin D and its synthetic analogues Ro-25-9022 and Ro-26-2198, and all-trans-, 9-cis-, and 13-cis-retinoic acid (Figure 5

). All vitamin D compounds significantly increased ALA–PpIX accumulation in LNCaP cells. Vitamin D was active at 10 and 100 nM, while the derivatives showed similar efficacy at 10-fold lower concentrations (Figure 5). Retinoid treatment even at 10 μM induced only moderate ALA–PpIX increases that did not reach statistical significance.

Finally, the vitamin D analogue Ro-26-2198 was examined for its effect on ALA-dependent photosensitisation. Pretreatment of LNCaP cells with Ro-26-2198 resulted in significantly increased phototoxicity (Figure 3D).

## DISCUSSION

This report demonstrates that a combination of differentiation therapy (DT) and PDT may be useful for enhancing cytotoxic effects on cancer cells. ALA-induced protoporphyrin IX (PpIX) production was markedly increased in LNCaP cells after treatment with differentiating agents such as R1881, and analogues of vitamin D, resulting in significantly enhanced phototoxicity in the differentiated cells. This was in contrast to many reports showing higher PpIX levels in more rapidly proliferating cells but confirmed our own findings in an earlier report (Ortel *et al*, 1998, and references therein). While the photodynamic sensitivity was slightly decreased in differentiated LNCaP cells as compared to controls (see below), enhanced PpIX production far outweighed the small reduction of PDT efficacy in DT-pretreated cells. Perhaps our most important finding, from a therapeutic standpoint, is the demonstration that increased PpIX levels result not from very large increases in a few cells, but rather from a more-or-less uniform increase of PpIX in many cells across the board. Thus, our flow-cytometry data (Figure 4) reveals that differentiating agents tend to shift a majority of cells above the threshold for cell-killing. This population effect can be appreciated from the fact that fluences necessary to achieve 50% cytotoxicity in undifferentiated cells, are more than 50 times higher than those required in differentiated (R1881-pretreated) cells.

Normal regulation of proliferation, differentiation, and apoptosis is lost in cancer cells (Andreeff *et al*, 2000). Because these three aspects are interconnected (i.e., arrest of the cell-cycle is a prerequisite for differentiation, and differentiation ultimately results in cell death in many systems (Sorrentino *et al*, 1990; Umek *et al*, 1991; Morioka *et al*, 1998; Gandarillas *et al*, 1999; Zermati *et al*, 2000), DT exploits these associations and restores pathways that have been apparently lost during carcinogenesis. The typical clinical paradigm of DT, epitomized by all-trans-retinoic acid (ATRA) treatment of APML, requires months of pharmacological therapy. ATRA induces maturation of the cancer cells along the granulocytic lineage (Breitman *et al*, 1981) and eventual apoptosis. ATRA is efficient in new and relapsed APML, but may induce resistance to further ATRA therapy (Dore *et al*, 1994). We approach DT from a different perspective. In our combined regimen, DT does not aim at persistent cellular differentiation, growth arrest, and apoptosis but rather exploits short-term cellular alterations in response to androgens, retinoids, or vitamin D. While a short course of DT may not have any long-term therapeutic effect on its own, unwanted long-term side effects (e.g. vitamin D effects on calcium metabolism) may be largely prevented by the brevity of exposure to the differentiating agent.

The success of our approach, using cellular differentiation to modulate ALA-PDT, depends upon the availability of suitable differentiation-modulating agents that work in the tumor of interest. We began these studies using R1881, a well-established modulator of androgen-responsive LNCaP cells (Esquenet *et al*, 1997). R1881 induced growth arrest and differentiation in LNCaP cells, as demonstrated by reduced DNA synthesis and expression of the molecular markers p27/Kip 1 and E-cadherin (Figure 1). Complete terminal differentiation, however, may not be a prerequisite for enhanced ALA-PDT. Perhaps only a few neoplastic cell types will undergo a complete differentiation program, but as long as PpIX formation is enhanced to therapeutic levels by the DT, then the objective of the combined regimen will have been achieved. By the same token, differentiation may not enhance PpIX formation in all cells or tumour types. This was demonstrated in the promyelocytic leukemia cell line HL-60, in which DMSO-induced differentiation did not result in increased PpIX production (Li *et al*, 1999). Similarly, larger amounts of PpIX were accumulated in lectin-stimulated lymphocytes, than in non-stimulated, non-dividing cells (Rittenhouse-Diakun *et al*, 1995). These observations illustrate that not all cell-types may be amenable to DT as a way to augment the efficacy of ALA-PDT, and that the cell type along with the cell's baseline heme-synthetic capacity, may determine PpIX accumulation after induction of differentiation.

An association of differentiation and increased ALA–PpIX formation was first described in an erythroleukemia cell line, in which increased heme synthesis is an integral part of the erythrocyte-specific differentiation program (Malik *et al*, 1989). In differentiated primary mouse keratinocytes several factors were shown to contribute to elevated cellular PpIX levels, including increased ALA uptake and enhanced PpIX production (Ortel *et al*, 1998). Here in LNCaP cells, as in primary keratinocytes (Ortel *et al*, 1998), the large increases of PpIX in differentiated cells resulted in strongly increased phototoxicity compared with non-differentiated cells exposed to the same ALA concentration (Figure 3).

Several potential caveats to our conclusions should be mentioned. First, to evaluate the inherent metabolic sensitivity of proliferative *vs* differentiated cells to ALA-PDT injury, we irradiated undifferentiated and differentiated cells that contained similar levels of PpIX (to determine photodynamic efficiency), and found that DT actually decreased the inherent cellular sensitivity to PDT. While the difference was statistically significant, it was much smaller than the selective enhancement of PpIX production in differentiated *vs* proliferating cells incubated at the same ALA concentration. Secondly, androgens are unlikely candidates for clinical use in prostate cancer, because of the risk of encouraging growth in certain cell clones. Therefore, it is encouraging that the activity of vitamin D and its synthetic analogues Ro-25-9022 and Ro-26-2198 bears promise for use in *in vivo* protocols. The high efficiency of the vitamin D analogues at low concentration, and the short course of DT required, make potential effects of these agents on calcium homeostasis a relatively minor concern (Shiohara *et al*, 2001). It remains to be determined, how specific short-term DT regimens will affect tumour biology and other factors in ALA-dependent PDT *in vivo*. These factors include local ALA concentration, and homogeneity of PpIX production, both important in the assessment of clinical applicability *in vivo*. However, the capability of DT to strongly enhance the efficacy of PpIX formation at all ALA concentrations studied may help to make future clinical protocols more efficient, less toxic, or both.
